# Regenerative potential of concentrated growth factor compared to platelet-rich fibrin in treatment of necrotic mature teeth: a randomized clinical trial

**DOI:** 10.1038/s41405-024-00288-3

**Published:** 2025-02-03

**Authors:** Taghreed Salah, Wael Hussein, Heba Abdelkafy

**Affiliations:** Department of Endodontics, Faculty of Dental Medicine for Girls, Al-Azhar University, Cairo, Egypt

**Keywords:** Root canal treatment, Pulp conservation

## Abstract

**Aim:**

This study aimed to compare the potential of two regenerative endodontic procedures, platelet-rich fibrin (PRF) and concentrated growth factor (CGF), in the treatment of mature permanent teeth with necrotic pulps and periapical radiolucency.

**Methods:**

This randomized clinical trial was written according to the Preferred Reporting Items for Randomized Trials in Endodontics 2020 guidelines. The study was registered in the Clinical Trials Registry (ClinicalTrials.gov) with identifier number NCT06227533. Eighteen patients with mature necrotic maxillary incisors with periapical lesions were randomly divided into two groups, one group received treatment with PRF (*n* = 9), while the other received CGF (*n* = 9). Radiographs were taken at the beginning, 6 and 12 months after treatment, and confirmed with CBCT to assess periradicular healing. An electric pulp tester and cold test were used to determine whether pulp sensibility had been regained during the follow-up period. The statistical analysis used an independent *t*-test to compare different groups. Repeated measures ANOVA to compare pre-operative, after 6 months, and after 1 year in the same group.

**Results:**

Both groups experienced a considerable increase in healing at 6 and 12 months compared to baseline, with no significant differences seen regarding lesion size (*p*-value was 0.34) and relative bone density (*p*-value was 0.27). There was no substantial change in the tooth sensibility reading between both groups at 6 months (*p*-value = 0.34), After 1 year, both groups exhibited identical patterns.

**Conclusion:**

Revascularization using PRF or CGF successfully preserved mature teeth with necrotic pulps.

## Introduction

Conventional root canal therapy (RCT) has long been used to preserve natural dentition in mature permanent teeth with necrotic pulps, guaranteeing both function and appearance. RCT involves cleaning and shaping the root canal system, administering medication, and obturating with an inert, biocompatible material. However, this method does not restore pulp tissue vitality, leads to loss of protective mechanisms, and promotes tooth brittleness [1]. Moreover, discoloration and loss of natural translucency of teeth are usually observed because of the loss of moisture inside the root as a result of the removal of the pulp tissue [2].

Endodontists are increasingly focused on utilizing natural materials over synthetic alternatives, particularly with the growing integration of tissue engineering across medical and dental disciplines. Endodontics, in particular, offers numerous applications for tissue engineering, including dentin–pulp complex regeneration, periodontal regeneration, and bone healing and regeneration. Regenerative endodontic procedures, a key application of tissue engineering, were initially developed as an alternative to mineral trioxide aggregate (MTA) apexification for immature teeth. However, these procedures have recently been expanded to include mature teeth as a viable alternative to conventional endodontic treatment [3].

Regenerative endodontic procedures (REPs) are defined as biologically based approaches aimed at restoring damaged structures, such as dentin, root structures, and cells of the pulp–dentin complex. The success of REPs relies on the coordinated interaction of the tissue engineering triad: stem cells, which are undifferentiated cells capable of continuous division and differentiation into various tissue types; growth factors that signal cellular activities; and scaffolds that provide structural support to guide the formation of the desired tissue [4, 5]. Its degradation rate aligns with the pace of tissue formation. A high porosity and optimal pore size support efficient cell seeding and enable the diffusion of cells and nutrients throughout the entire structure [6]. The literature has reported the use of platelet-rich plasma (PRP), platelet-rich fibrin (PRF), collagen, blood clots, and other naturally occurring scaffolds are examples of autologous scaffolds [7–9].

Blood clots are considered the simplest approach to form a scaffold that forms within a properly disinfected root canal space by intentionally inducing bleeding from the periapical tissue. However, the blood clots have their limitations as achieving periapical bleeding in the canal space is not always feasible. PRP serves as a natural scaffold, reducing the risk of cross-infection and immunogenicity. However, it is not entirely autologous [10]. PRP releases growth factors quickly, typically within 7–14 h, after which their levels begin to decline [11].

Platelet-rich fibrin (PRF) was introduced in regenerative endodontic procedures (REPs) to address the limitations of PRP and blood clots. PRF not only enhances the release of growth factors and leukocytes but also offers a completely autologous composition. Its preparation requires no additional thrombin, as PRF forms through a gradual polymerization process that integrates a higher concentration of cytokines into the fibrin matrix [11]. This structure allows for the sustained release of growth factors over 7–14 days. PRF is also cost-effective and easy to prepare, making it a practical choice in clinical settings.

Recently, concentrated growth factor (CGF) has been introduced, unlike PRP, the production of CGF is additive-free and follows a simpler protocol that involves varying the centrifugation speed. This process yields a fibrin network closely resembling natural tissue, enriched with growth factors and proteins derived from autologous platelets and leukocytes. Known as a bioscaffold and cytokine reservoir, CGF represents the latest generation of platelet concentrate products, widely used for bone regeneration in implantology [12]. CGF contains high levels of essential growth factors, including platelet-derived growth factor-BB (PDGF-BB), transforming growth factor β-1 (TGF-β1), insulin-like growth factor-1 (IGF-1), vascular endothelial growth factor (VEGF), and basic fibroblast growth factor (bFGF). These factors play crucial roles in regulating cell differentiation, proliferation, and angiogenesis, all vital for tissue regeneration [13].

CGF seems to have better therapeutic applications as a biomaterial and cytokine combo product to promote pulp regeneration. Therefore, the current investigation aimed to assess radiographic healing and the potential for pulp sensibility to be restored in adult permanent teeth that had necrotic pulps that had been treated with CGF and PRF. The null hypothesis stated that the evaluated groups would have no discernible statistical difference in periapical healing and recovering sensation.

## Subjects and methods

### Study design

The study was conducted after obtaining the approval of the research ethics committee (REC) of the Faculty of Dental Medicine, Al-Azhar University, with the primary code (PEN-21-02) and the final code (REC-PD-24-01). The study was also registered in the Clinical Trials Registry (ClinicalTrials.gov) with identifier number NCT06227533. This study was designed as a double-blinded (patients and assessors) randomized controlled clinical trial with two-arm parallel groups with an allocation ratio of 1:1. It was carried out in the endodontic department clinic at the Faculty of Dental Medicine, Al-Azhar University. This study followed the Consolidated Standards of Reporting Trials (CONSORT) guidelines (extension to randomized pilot and feasibility trials) Preferred Reporting Items for Randomized Trials in Endodontics (PRIRATE) 2020 guidelines [14, 15].

### Sample size

To conduct a statistical test of the null hypothesis “which states that pulp sensibility in necrotic adult single-rooted teeth with periapical radiolucency is the same” a power analysis was constructed utilizing PRF and CGF. The expected sample size (*n*) was determined to be (18 root canal) instances by using an alpha level of (0.05) and a beta of (0.2), meaning power = 80% and effect size (*d*) of (1.4) computed based on the findings of Nageh et al. [16]. The G*Power version 3.1.9.7 informed consent form, which contained details on the goal of the study and the benefits and drawbacks of the treatment, was used to calculate the sample size [17].

### Patients’ selection

For this investigation, 36 teeth in a total of 28 patients were investigated. Only 18 mature teeth diagnosed with pulp necrosis and periapical lesions were included in the study following clinical and radiological evaluation.

The inclusion criteria and exclusion criteria were as follows [18]: participants were aged between 18 and 30 had no sex preferences, free of systemic disorders, and were prepared to give their informed consent. According to Ørstavik et al.‘s categorization [19], they also need to have necrotic single-rooted maxillary incisors with developed roots and periapical lesions with periapical index (PAI) scores ≥3. The tooth should also have been restorable with a direct coronal restoration. Pregnant women, those with widespread chronic periodontitis, teeth that cannot be restored, pulp space needed for post-cementation, teeth that cannot hold a rubber dam, root canal therapy in the past, developmental abnormalities, and external or internal resorption were among the exclusion criteria. Every patient carefully read and signed an informed consent form that detailed the goals of the trial as well as the benefits and drawbacks of the treatment.

### Randomization and blinding

Each of the 18 sheets per tooth numbered 1 through 18, is carefully packaged in an opaque envelope. An envelope was given to each patient before the start of the second visit. The participant’s regenerative protocol was determined by the number found in the envelope. The patient was blinded by the technique to which they were submitted.

### Patient diagnosis

Accurate case diagnosis, taking into account the patient’s dental and medical background. Following the guidelines on the endodontic clinical diagnostic sheet, a comprehensive examination of the tooth and its supporting tissues was carried out. To assess the amount of periapical radiolucency, periodontal status, and, if present, root resorption, a preoperative periapical radiograph was taken using the standardized paralleling technique with an intraoral sensor (V sensor, RVG, Eighteeth company, China) and digital X-ray system (2200 Kodak Dental Systems, Rochester, intraoral X-ray system, USA). Pulp sensitivity was measured numerically using an electric pulp tester (Denjoy Dental Co.‘s Pulp Tester DY 310). After drying and isolating the studied tooth, a small amount of toothpaste was administered to it. A lip clip was applied, and the test was carried out. The average of three readings at a 5-min interval was taken for each tooth. Pulp sensibility was also assessed using a thermal test (Endo ice) to identify the pulp state and compare it to the contralateral normal tooth.

### Intervention

The most recent operating guidelines for regenerative endodontic therapy (RET), published by the European Society of Endodontics (ESE) [20] and the American Academy of Endodontics (AAE) [21], state that two treatment visits are necessary for the standardized RET technique.

#### First appointment

All of the patients in this study had the same first appointment. The first appointment comprised an anesthetic with 1.8–3.6 mL of 2% lidocaine and 1:100,000 epinephrine (septodont, USA). Following isolation with a rubber dam, a straight-line access was created. The working length was clinically determined with an electronic apex locator (E PEX Pro, Eighteeth, China) and radiographically verified with an intraoral periapical radiograph. The root canal was constructed using Protaper Next files (DENTSPLY, Maillefer, Switzerland) up to size X3, and 1.5% NaOCl (Clorox Co., Ramadan 10th) was used as irrigation between each successive file, with a 30 G side vent needle (Ultradent) positioned ~2 mm from the root end.

**Fig. 1:**
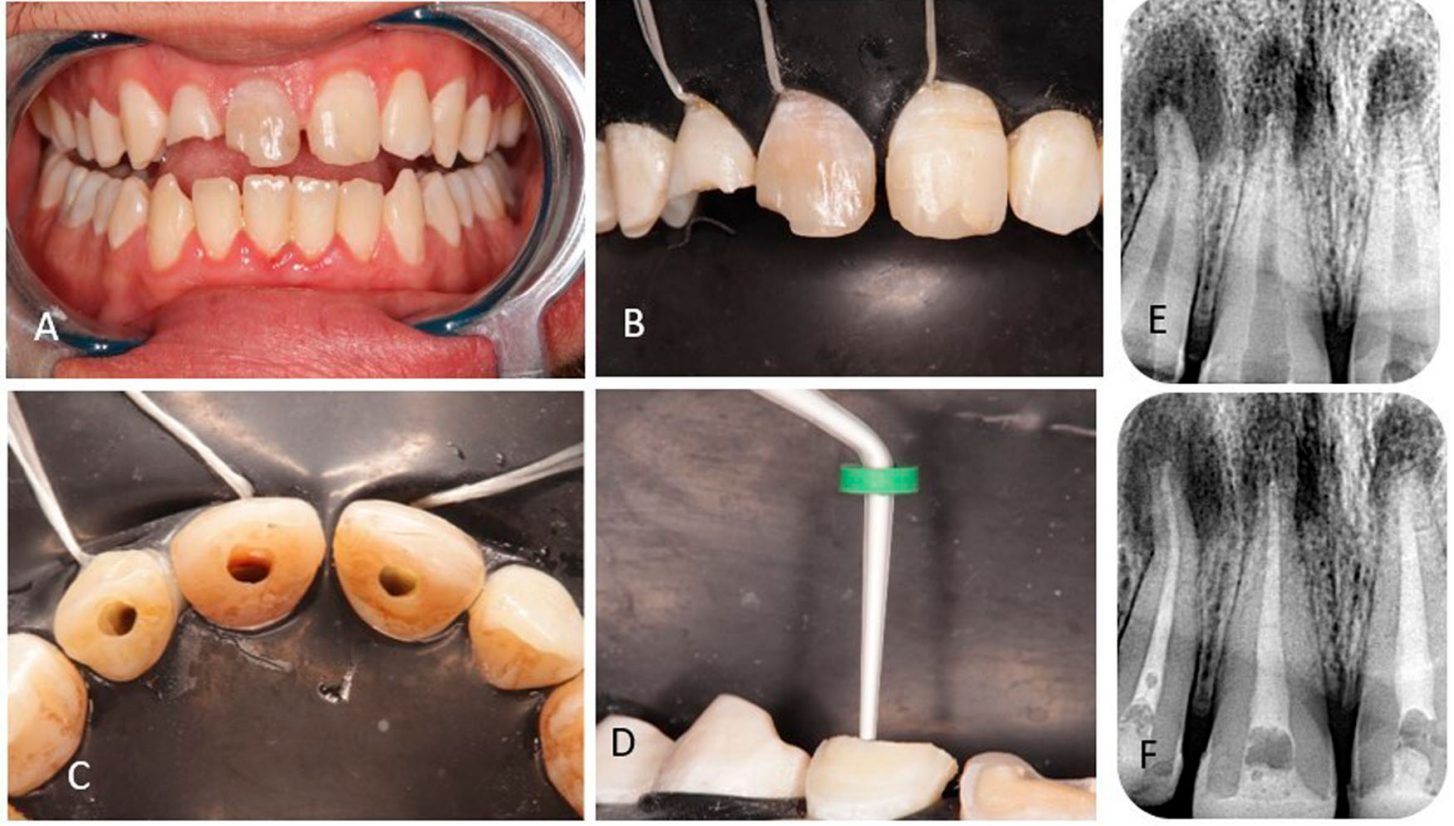
A photograph showing; The first appointment comprised an anesthetic. Following isolation with a rubber dam, a straight-line access was created. The root canal was constructed up to size X3, with the assigned irrigation protocol. Ultracal XS calcium hydroxide was administered. Glass Ionomer was used to temporize the teeth until the next visit. **A** initial situation, **B** isolation, **C** access preparation, **D** calcium hydroxide application, **E** pre-operative radiograph, and **F** radiograph showing calcium hydroxide in the canals.

Final irrigation once preparation was finished was 20 mL 1.5% NaOCl for 5 min, then 5 mL sterile saline solution (Al mottahedoon pharma, Egypt) for 5 min, and lastly 20 mL 17% EDTA (Calix E, Dharma, USA) for 1 min [22]. Canals were subsequently dried using paper points X3 (Dia-proT Next, Diadent) and, in accordance with the manufacturer’s recommendations, Ultracal XS calcium hydroxide (Ultracal XS, Ultradent, USA) was administered 2 mm below the radiography apex. After radiography was used to evaluate the calcium hydroxide quality, Glass Ionomer (Kavitan Plus, Kerr, USA) was used to temporize the teeth until the next visit. Two weeks later, patients were summoned back (Fig. 1).

#### Second appointment

Patients came back for a follow-up appointment after 2 weeks. The calcium hydroxide was reinserted for a further two weeks if the symptoms persisted. Patients who do not exhibit any symptoms move on to the following RET therapy steps: The procedure involved applying local anesthesia without the use of a vasoconstrictor (3% Mepecaine; Alexandria Pharmaceutical), field isolation with a rubber dam, and temporary seal removal. After flushing out the calcium hydroxide with 5 mL of saline, the canals were irrigated with 20 mL of 17% EDTA for 5 min, which was activated for 1 min with an E1 ultrasonic tip (Woodpecker Ultrasonic), and then with sterile saline solution. The canal was dried with sterile paper points. Then the regeneration process was conducted based on the patient’s group. Over-instrumentation and rotation of a slightly pre-curved K-file (#35 size, Mani Inc., Japan) at 2 mm past the apical foramen resulted in bleeding in the canal system, which was thereafter filled with blood to the level of the CEJ.

### Group I: platelet-rich fibrin (PRF) (*n* = 9)

**Fig. 2:**
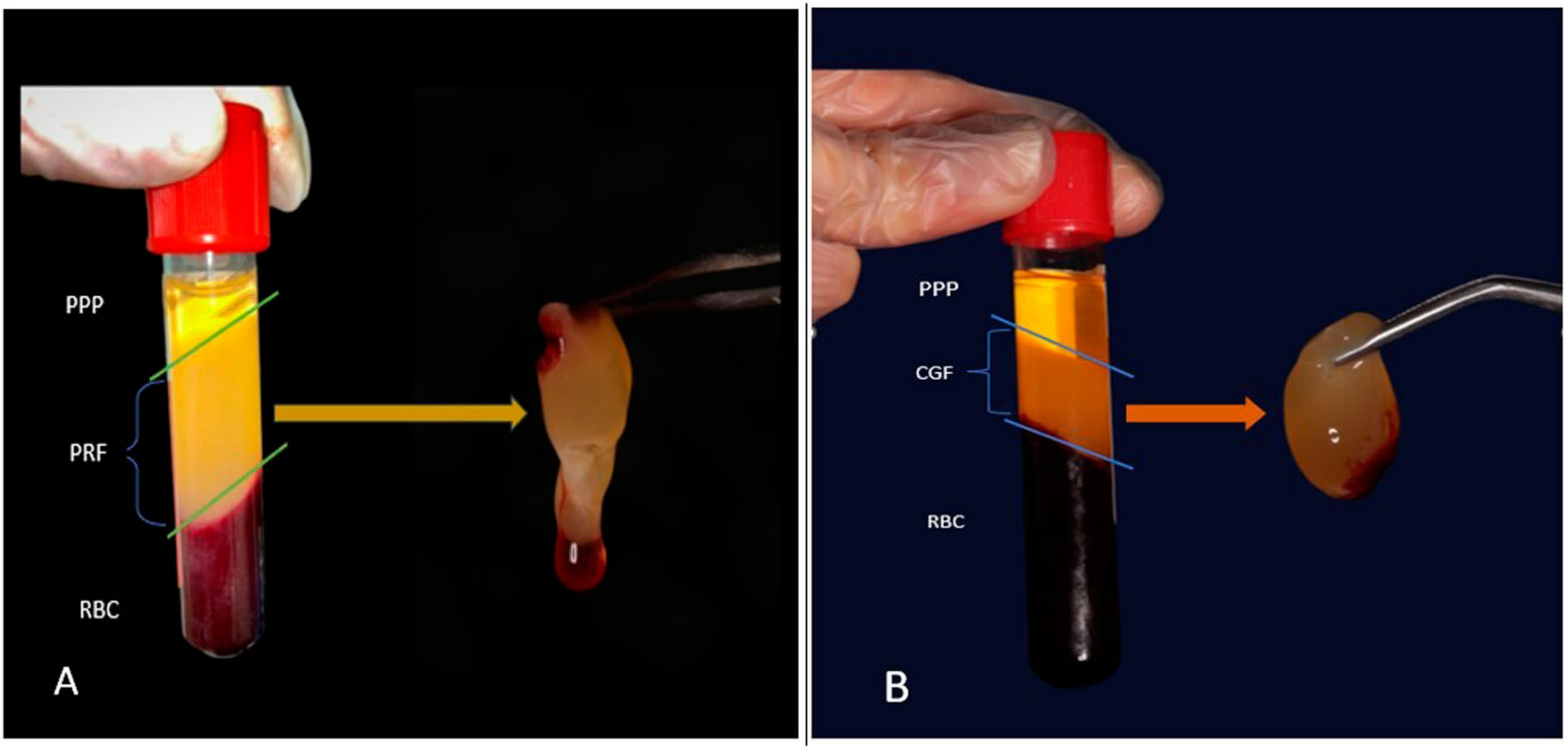
A photograph showing; PRF preparation: A 5 mL sample of blood was drawn from the patient’s and centrifuged at 3000 rpm (400×g) for 10 min in a test tube without anticoagulant at room temperature. The finished result had three layers: PRF made up the middle layer. CGF preparation: A total of 10 mL of venous blood was collected. The tubes were centrifuged using a one-step centrifugation protocol at variable rpm, 2 min at 2700 rpm (600×g), 4 min at 2400 rpm (400×g), 4 min at 2700 rpm (600×g), 3 min at 3000 rpm, and 36 s deceleration. The final product consisted of three layers: CGF filled the middle layer. **A** platelet-rich fibrin and **B** concentrated growth factor (CGF).

PRF preparation: A 5 mL sample of blood was drawn from the patient’s right median cubital vein into a glass tube and centrifuged at 3000 rpm (400×*g*) for 10 min in a test tube without anticoagulant at room temperature using a tabletop fixed angle centrifuge (80-2 Electronic Laboratory Medical Centrifuge, Jiangsu, China**)** [23]. The finished result had three layers: red blood cells occupied the bottom layer, platelet-rich fibrin made up the middle layer, and serum filled the top layer (Fig. 2A). The PRF was separated using a sterile scissor and compressed into a membrane, which was then fragmented and placed into the canal gradually until it reached the CEJ level using a hand plugger and a finger spreader #40.

### Group II: Concentrated growth factor (CGF) (*n* = 9)

A total of 10 mL of venous blood was collected and transferred to sterile glass tubes without an anticoagulant solution. The tubes were centrifuged at room temperature in a medifuge machine (Silfradent, Italy) using a one-step centrifugation protocol at variable rpm, which consists of 2 min at 2700 rpm (600×*g*), 4 min at 2400 rpm (400×*g*), 4 min at 2700 rpm (600×*g*), 3 min at 3000 rpm, and 36 s deceleration [23]. The final product consisted of three layers: serum filled the top layer, CGF filled the middle layer, and red blood cells filled the bottom layer (Fig. 2B). After being separated from the red blood cells, the concentrated growth factor-containing fibrin gel was thoroughly squeezed to form a membrane. Following the induction of bleeding in the canal, as observed in group I, the newly prepared CGF membrane was fragmented and stuffed into the canals up to the cementoenamel junction using a plugger (sedradent, Egypt).

**Fig. 3:**
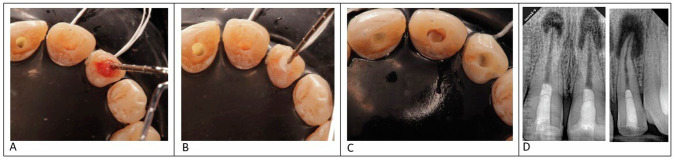
A photograph showing: in a second appointment after placement of PRF or CGF, resorbable collagen matrix was placed, then a 3 mm MTA plug was placed at the level of CEJ, and the coronal cavity was sealed using a layer of self-adhering flowable composite. And the final filling using composite. **A** insertion of the scaffold into the canal, **B** insertion of resorbable collagen matrix, **C** after the placement of mineral trioxide aggregate (MTA) and **D** immediate post-operative radiograph.

For both groups, resorbable collagen matrix (Hemospon, Maquira, Brazil) was placed, then a 3 mm MTA plug (Retro MTA, BioMta, South Korea) was placed at the level of CEJ, and the coronal cavity was sealed using a layer of self-adhering flowable composite (Kerr Dyad Flow, Kerr, CA, USA). Plus layer as a cavity liner, and the final filling using composite. (Beautifil II, Shofou, Japan) (Fig. 3).

Follow-up: At 6 and 12 months, patients were recalled to examine their treated teeth using an electric pulp tester and thermal test to ensure pulp responsiveness was restored. To examine periapical healing, use a digital radiograph. CBCT images were taken at baseline and 12 months following therapy in selected cases.

## Methods of evaluation

### Size of periapical lesion

The size of the periapical lesion was known by encircling the margins of the periapical lesion and drawing 2 straight lines from the margins passing through the center, the vertical one represents length while the horizontal one represents width. Then the area of the oval shape was calculated according to the equation (*π* × *r*1 × *r*2 (*r*1 = ½ length, *r*2 = ½ width))

Healing of periapical tissues was evaluated by comparing the change in area of the periapical lesion in preoperative, 6-month, and 1-year follow-up radiographs.

### Relative bone density

The relative bone density is calculated by comparing the mean grey value of the bone defect and the healthy surrounding bone that does not overlap with other anatomic features. To form a quotient, use the following formula:

$${{{{\rm{Relative}}}}\; {{{\rm{bone}}}}\; {{{\rm{density}}}}}=\frac{{{{{\rm{Mean}}}}\; {{{\rm{grey}}}}\; {{{\rm{value}}}}\; {{{\rm{of}}}}\; {{{\rm{the}}}}\; {{{\rm{defect}}}}\; {{{\rm{region}}}}}}{{{{{\rm{Mean}}}}\; {{{\rm{grey}}}}\; {{{\rm{value}}}}\; {{{\rm{of}}}}\; {{{\rm{the}}}}\; {{{\rm{surrounding}}}}\; {{{\rm{bone}}}}}}$$

The measurement of the mean grey values was performed with the software ImageJ 1.44p (Wayne Rasband, National Institute of Health, USA) [24].

Comparing the relative bone density which indicates remineralization of bone in preoperative, 6-month, and 1-year follow-up radiographs to evaluate the periapical healing process.

### Sensation

The preoperative results of the thermal and electric pulp testers were noted to verify whether the concerned tooth had pulp necrosis or not. To find out if the teeth in the study regained sensitivity or not, test results from 6-month and 1-year follow-ups were analyzed.

#### Outcomes [20]

##### Primary outcome

The elimination of symptoms and the evidence of bony healing.

##### Secondary outcome

Positive response to vitality testing (which if achieved, could indicate a more organized vital pulp tissue).

### Statistical analysis

Statistical analysis was performed with SPSS 20®, Graph Pad Prism®, and Microsoft Excel 2016. All quantitative data were presented as minimum, maximum, mean, and standard deviation. All qualitative data were presented as Frequency and percentages. Data were checked for normality using the Shapiro–Wilk and Kolmogorov test. Independent *t*-test to compare between two different groups. Repeated measures ANOVA to compare between pre-, after 6 months, and after 1 year in the same group. The Chi-square test and Fisher’s exact test were used to compare qualitative data.

## Results

**Fig. 4:**
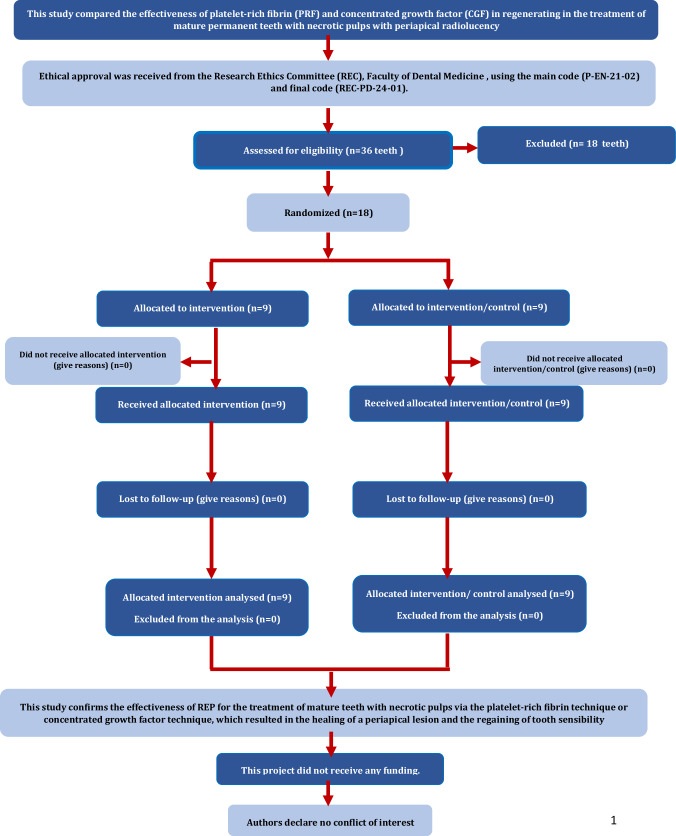
The consolidated standards of reporting trials flowchart of the research design.

The research design flowchart for the Consolidated Standards of Reporting Trials is shown in Fig. 4.

### Radiographic evaluation (healing)

#### Lesion size

**Fig. 5:**
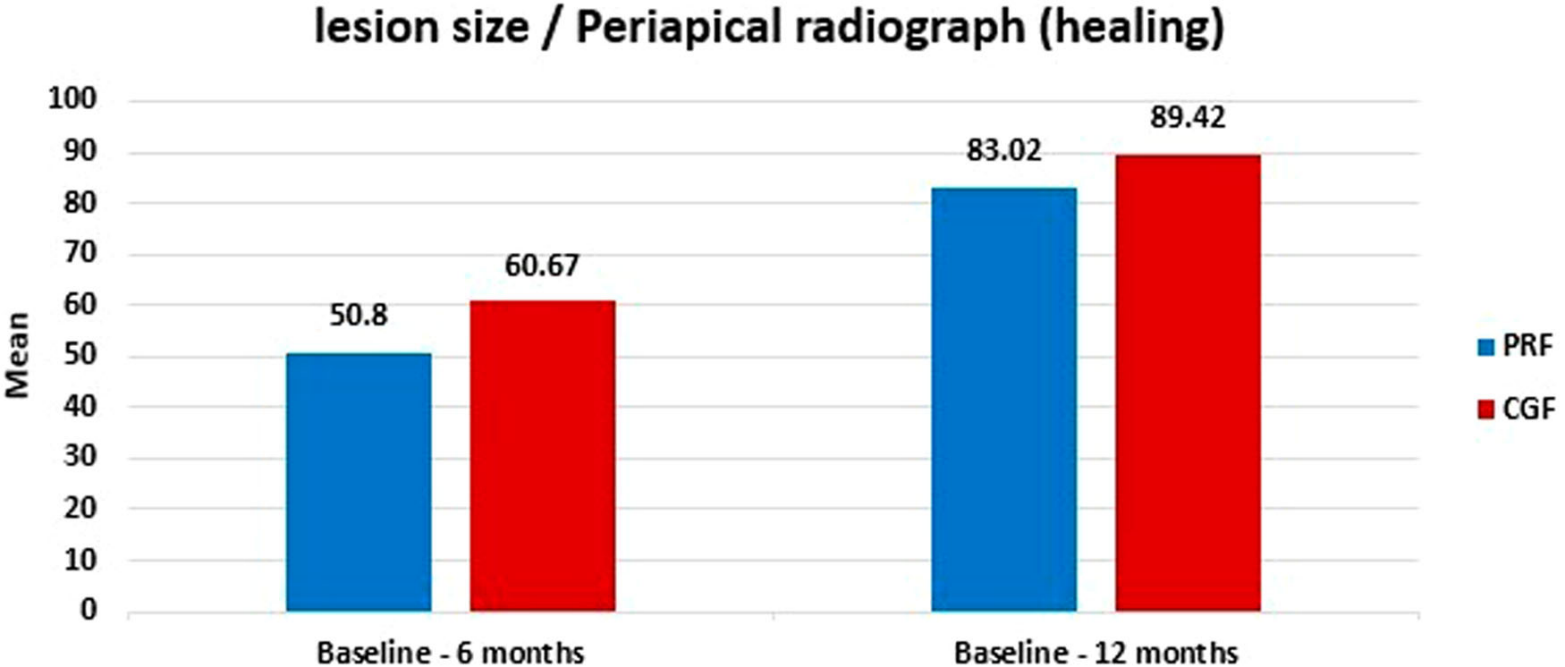
Bar chart showing lesion size regarding periapical radiograph (healing) in group I (PRF) and group II (CGF).

A comparison of the percentage of healing regarding changes in the periapical lesion (reduction in size) between Group I (PRF) and Group II (CGF) (Fig. 5). Regarding the results of baseline—after 6 months, the PRF group was (50.80 ± 16.85) and the CGF group (60.67 ± 11.76) with a difference between them (−9.88 ± 6.85), while from baseline—12 months: the PRF group (83.02 ± 17.13) and the CGF group (89.42 ± 9.22), with the difference (−6.40 ± 6.49). Indicating that the difference was statistically insignificant at both follow-up periods between the two groups (*p*-value = 0.14, 0.27) respectively. However, within each group, there was a statistically significant difference in both follow-up periods *p*-values for the PRF group (0.0002) and CGF group (0.0008).

**Fig. 6:**
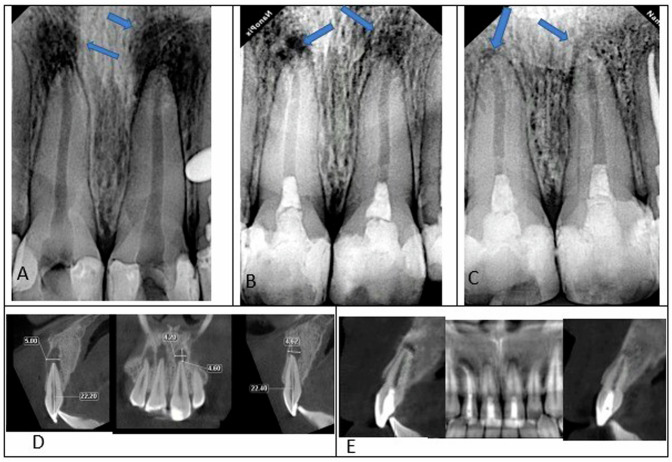
Radiographs of fully grown maxillary central incisors treated with regenerative endodontic procedures (REPs) utilizing a platelet-rich fibrin-based method with periapical radiolucency. **A** A preoperative periapical radiograph. **B** The radiograph was obtained 6 months later. **C** Following a year of monitoring, the radiograph demonstrates significant progress in the periapical lesion confirmed by CBCT at baseline (**D**) and 12 months follow-up (**E**).

**Fig. 7:**
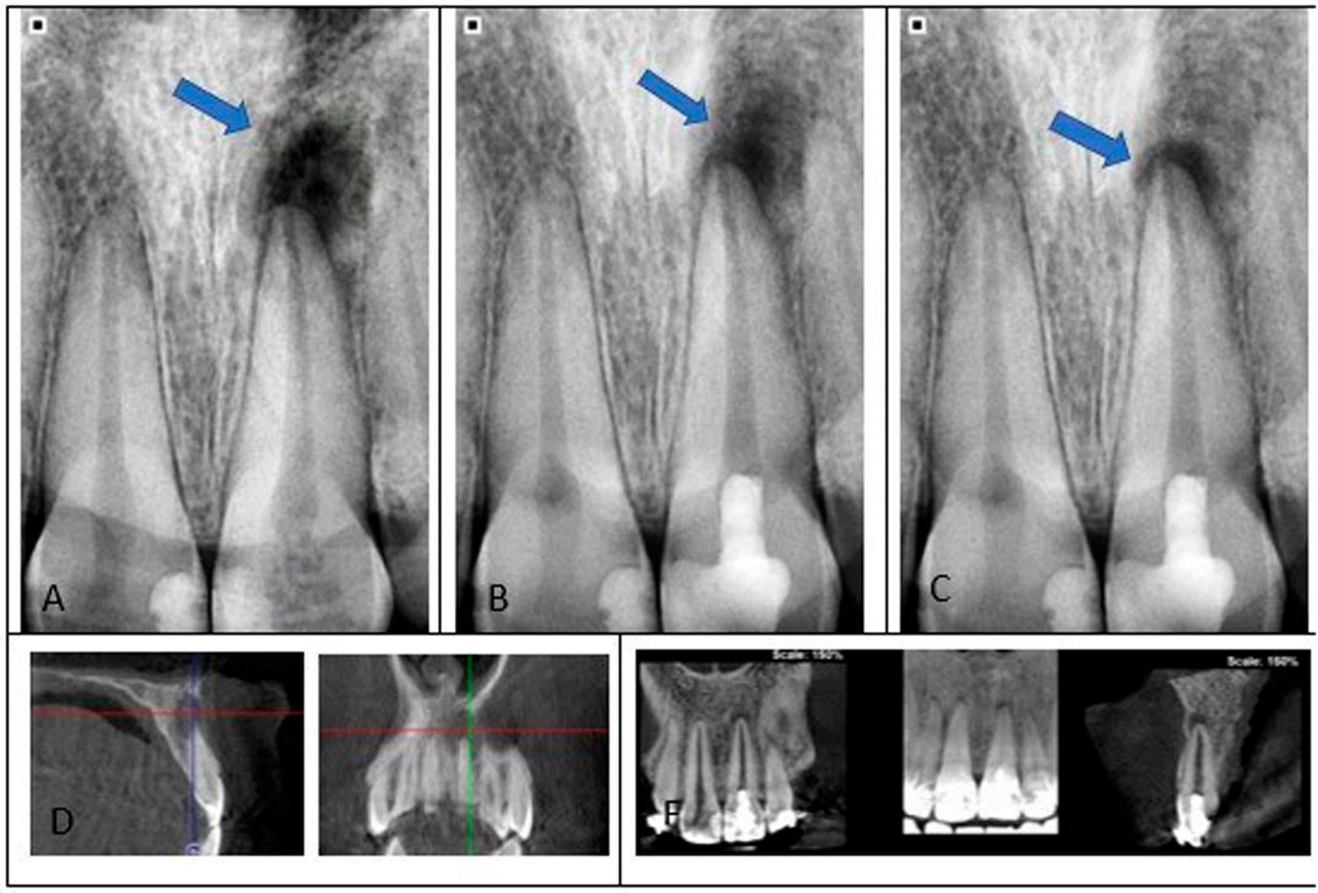
Radiographic images of a mature maxillary central incisor that underwent periapical radiolucency treated with concentrated growth factor-based regenerative endodontic procedures (REPs). **A** A preoperative periapical radiograph. **B** The radiograph was obtained 6 months later. **C** The radiograph demonstrates significant recovery in the periapical lesion following a 12-month follow-up confirmed by CBCT at baseline (**D**) and 12 months follow-up (**E**).

Variations in the radiographic lesion size between the tested groups throughout the follow-up are shown in Figs. 6 and 7.

#### Relative bone density

**Fig. 8:**
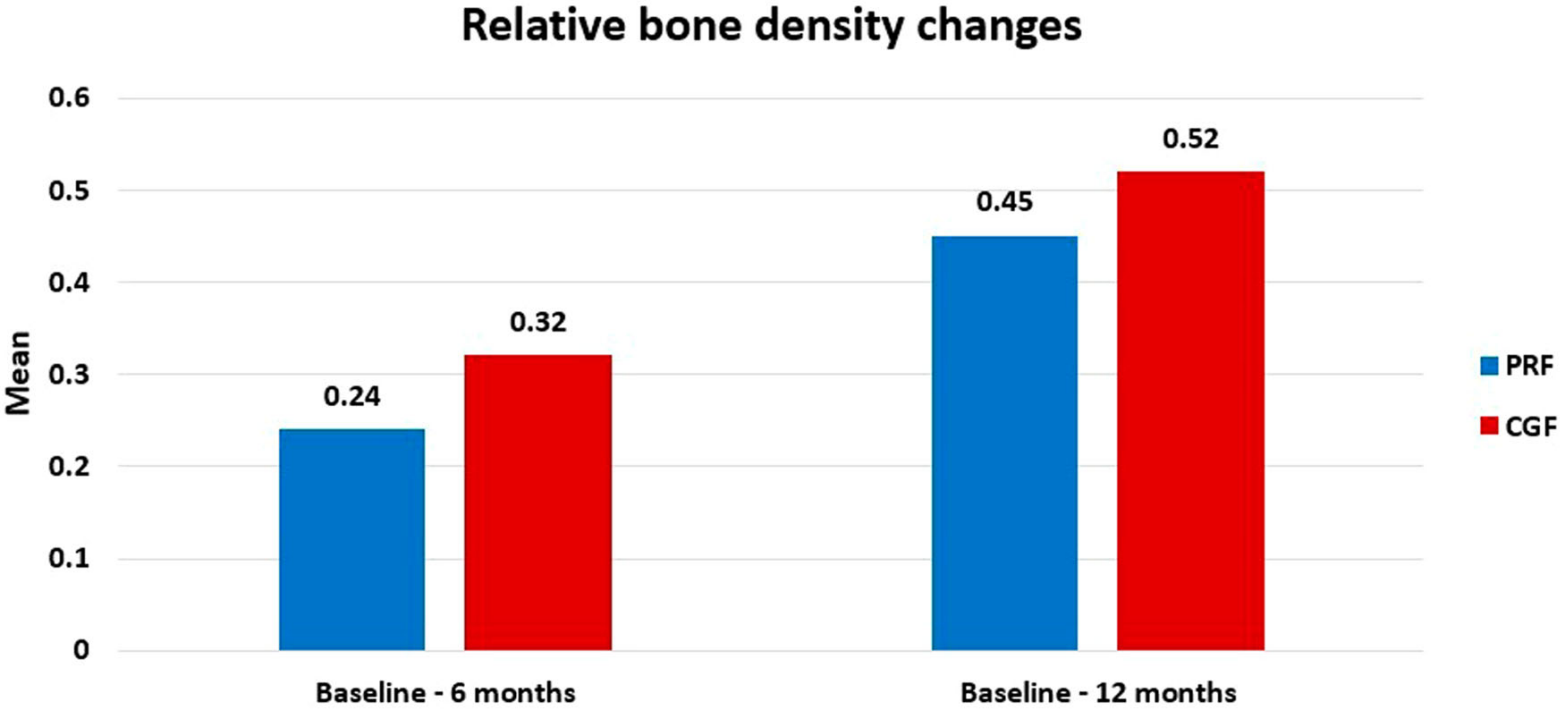
Bar chart showing relative bone density changes in group I (PRF) and group II (CGF).

A comparison of the percentage of healing regarding changes in the relative bone density between Group I (PRF) and Group II (CGF) (Fig. 8). Regarding the results of baseline—after 6 months, the PRF group was (0.24 ± 0.11) and the CGF group (0.31 ± 0.11) with a difference between them (−0.08 ± 0.05). while from baseline—12 months: the PRF group (0.45 ± 0.15) and the CGF group (0.52 ± 0.08), with the difference (−0.06 ± 0.06). Indicating that the difference was statistically insignificant at both follow-up periods (*p*-value = 0.14, 0.27) respectively. However, within each group, there was a statistically significant difference in both follow-up periods *p*-values for the PRF group (0.0003) and the CGF group (0.0003).

### Pulp sensibility

A result between 1 and 39 indicates a tooth having vital pulp, according to the manufacturer’s recommendations for the electric pulp test. A result between 40 and 79 indicates that some of the dental nerves are nonvital. Finally, if the values were 80, it showed that the tooth had necrotic, nonvital pulp. The 100% frequency of “No” for both groups indicates that none of the patients in either group reported any feeling about the EPT or cold test before the therapy. In all groups, the proportion of teeth that regained sensation grew steadily after treatment and peaked at 12 months (Table 1).

**Table 1:** Frequency and percentages of sensation at pre-, after 6 months, and after 1 year in group I (PRF) and group II (CGF).

			PRF		CGF		*P* value
			*N*	%	*N*	%	
Sensation	Pre	No	9	100.0	9	100.0	…..
		Yes	0	0.0	0	0.0	
	6 months	No	5	55.6	3	33.3	0.34
		Yes	4	44.4	6	66.7	
	1 year	No	3	33.3	3	33.3	1
		Yes	6	66.7	6	66.7	

## Discussion

The purpose of this study was to determine if two regenerative endodontic procedures (REPs)—CGF-based technique and PRF-based technique—were effective in reviving necrotic pulp in adult permanent teeth. Our findings imply that both REP strategies—PRF or CGF—might be effective means of treating mature teeth with necrotic pulp.

Root canal therapy, which includes chemo-mechanically preparing the root canal and closing it with a biocompatible material, has been used to treat mature necrotic permanent teeth [25]. Unlike traditional root canal treatment, REPs are primarily intended for immature permanent teeth with necrotic pulp. They focus on disinfection with minimal or no drilling, followed by encouraging bleeding within the canal. This bleeding forms a clot, which is then covered with a biocompatible material to aid healing [26]. REPs are a new approach being explored for mature teeth. They aim to address some limitations of traditional RCT, such as loss of sensation in the treated tooth, reduced immune response in the treated area, increased risk of cracks or fractures in the tooth [19], and potential for reinfection due to leaks around the filling [27].

While RCT focuses on removing infected tissue and sealing the canals, REP aims to regrow the pulp tissue itself. This approach could be beneficial for complex cases such as root fractures, perforating resorption, and even avulsion [28]. It has been suggested that REPs could potentially help re-establish the natural immune system within the tooth, potentially reducing the risk of reinfection in the root canal system [29] and may result in the restoration of tooth sensibility as well as a potential proprioceptive defense mechanism of the pulp, which act as a warning system for any damage to the tissue and shield it from more harm [30].

The irrigation process aimed to balance cleaning efficacy with promoting stem cell activity within the tooth. In this investigation, sodium hypochlorite served as the primary irrigant. Its ability to kill germs is associated with the production of hypochlorous acid when coming into contact with organic matter. To reduce the chance of irrigant extrusion into the periapical space, use a closed-end side-vented needle to perform extensive irrigation for five minutes at a total volume of 20 ml of 1.5% NaOCl [20]. While a high concentration (5.25%) of sodium hypochlorite (NaOCl) is a strong disinfectant and tissue remover, it is not ideal for promoting tooth regeneration. This concentration weakens the tooth structure by breaking down collagen [31] and offers minimal additional benefit in killing bacteria compared to a lower concentration (0.5%) [32]. Even worse, high-concentration NaOCl harms stem cells crucial for healing and hinders the formation of new dentin-forming cells [33]. Therefore, lower concentrations are preferred for procedures aiming to regenerate tooth tissue.

Following NaOCl, EDTA 17% for 5 min was used to counteract its negative effects and promote stem cell activity [34]. EDTA helps release growth factors from the dentin, which are beneficial for cell growth, movement, and development into dentin-forming cells [35]. Ultrasonic activation with EDTA was used to enhance the release of a specific growth factor (TGF-β1) from dentin [36]. Increased growth factors improve cell survival and movement, and act as a signal for stem cells (MSCs) from nearby tissues to migrate and differentiate into desired cells [37].

Calcium hydroxide was selected as the intracanal medicament between appointments due to its merits in regenerative endodontic procedures. It creates a high pH environment (12.5–12.8) that discourages bacterial growth [38], promotes the survival of stem cells in the apical papilla [39], and appears to increase levels of TGF-β1, a growth factor crucial for cell communication and differentiation within the dentin matrix [34].

In the second visit, the anesthetic used was 3% Mepivacaine without any vasoconstrictor. This was done to encourage bleeding into the canal system from the nearby tissues. To allow stem cells to migrate from the periapical area into the canal space, the apex was violated using K-file #35 [40].

PRF is a major improvement over PRP. Unlike PRP, PRF skips blood thinners and animal products, making it more natural. This natural process creates a strong, elastic scaffold that doesn’t break down quickly [41]. This scaffold offers a triple benefit: it allows cells to move in, stores important healing molecules, and slowly releases growth factors such as platelet-derived growth factor, transforming growth factor beta 1, fibroblast growth factor, and vascular endothelial growth factor from 7 to 28 days reaching the peak around day 14 and promote cell growth, specialization, and new blood vessel formation [42]. PRF’s advantage goes even further with the presence of white blood cells and other immune system elements, which can help control inflammation and fight infection [43].

CGF is an advanced type of second-generation of platelet concentrate. Developed by Sacco in 2006, CGF uses a unique centrifugation process with varying speeds (2400–2700 rpm) compared to PRF’s constant speed. This creates a denser matrix relatively stiffer than PRF or PRP, packed with more growth factors, offering sustained release for up to 2 weeks with a peak on day five.

The healing results of this study regarding periapical lesion size and relative bone density showed significant healing after one year in both treatment groups. This improvement might be the result of the natural wound healing process, which begins with the periapical tissue bleeding as the initial stage of the healing process [44]. Furthermore, REPs might improve antimicrobial clearance. The bleeding draws immune system components like cytokines, immunoglobulins, and phagocytes to the disinfected canal [45]. These components help locate and engulf bacteria, promoting healing. Additionally, REPs have been shown to stimulate bone repair in young permanent teeth [46] These findings align with other research on using REPs to treat necrotic teeth with apical periodontitis in mature adults [16, 18, 22, 47].

Unfortunately, there is a lack of data to compare periapical healing between the two procedures for revascularizing mature teeth. Several research have used both approaches for revascularizing immature teeth. The study found no significant difference (*p* < 0.05) between the PRF and CGF groups in terms of clinical performance, including remission of symptoms, periapical healing, and ongoing root development [21, 48].

This study found that measurements of periapical lesions decreased at the end of 12 months in both groups when compared to 6-month measurements. However, the CGF group had a better periapical lesion healing response than the PRF group after 6 and 12 months. This is because CGF has a denser fibrin matrix, which is richer in growth factors than PRF. A review by Li et al. supports this, highlighting CGF’s superior composition and effectiveness than its predecessors [49]. Additionally, a case report of revascularization of immature teeth using CGF published by Nivedhitha et al. demonstrated positive outcomes using CGF, including increased root thickness, apical foramen closure, and reduced periapical lesion size at the one-year follow-up [50].

A randomized clinical trial by Elheeny et al. found that CGF treatment significantly improved periapical healing compared to PRF treatment (*P* < 0.05). Clinically, both scaffolds had similar success rates (93.9%) across follow-up intervals [51].

In the present study, 66.7% of all the cases treated responded positively to cold after 12 months in both groups, and the same percentage responded positively to the EPT. This is in agreement with several studies that compared several techniques in the regeneration of mature teeth and showed there is no significant difference in regaining pulp sensibility after one-year follow-up [16, 22, 47, 51, 52].

However, the current investigation contradicted the findings of Saoud et al. [45] and Nagas et al. [53], who found negative responses to sensibility tests in adult teeth after 26 and 60 months of follow-up. In addition, Nassar et al. [54] reported that no teeth responded positively to EPT at the 12-month follow-up and only two teeth responded positively to the thermal test at the 12-month follow-up. They emphasized that a lack of pulp response does not necessarily reflect a lack of life. Cold and electric pulp testing may be affected by regenerated tissue levels and filling material thickness. This difference could be attributed to a change in the experimental design.

This study observed delayed responses to cold and electric pulp testing (EPT) in treated teeth compared to healthy teeth on the opposite side. However, these responses were positive, indicating the presence of vital tissue. This delay can be attributed to two factors: the coronal plug material and the developing nervous system within the regenerated pulp tissue. The plug material likely hinders the immediate perception of the pulp’s response. Additionally, the regenerated tissue itself may have an immature nervous system, leading to an initial delay. This explanation aligns with the finding that sensitivity responses increased over time, suggesting the development of a more mature nervelike tissue.

This initial trial had limitations, including a brief follow-up period. However, future studies will extend monitoring and follow-up to provide more robust data. As evidence accumulates on the successful use of REPs for treating necrotic pulp in mature teeth with various protocols, it could pave the way for specific guidelines and recommendations. This would mirror the ESE position statement for immature teeth revascularization procedures [21].

## Conclusion

This study, despite its limited sample size and follow-up period, produced promising results. PRF and CGF combined with a revascularization procedure successfully healed mature necrotic teeth with periapical lesions. After a year of follow-up, the platelet concentrates regimens successfully healed periapical lesions and restored sensitivity to necrotic teeth. The CGF group had a better healing response for the periapical lesion at 6 months compared to the PRF group, but there was little change at 12 months.

## Data Availability

The data is available from the corresponding author upon request.
